# Hsp90 co-chaperone FKBP4 facilitates CCT8 folding and connects Hsp90 to chaperonin-dependent proteostasis

**DOI:** 10.1016/j.jbc.2025.110914

**Published:** 2025-11-05

**Authors:** Yun-Yu Huang, Ya-Lan Chang, Yun Chen, Wei-Yu Chiang, Ai-Tao Chiang, Pang-Hung Hsu, Shu-Chun Teng

**Affiliations:** 1 Department of Microbiology, College of Medicine, National Taiwan University, Taipei, Taiwan; 2 Department of Bioscience and Biotechnology, National Taiwan Ocean University, Keelung, Taiwan; 3 Center of Precision Medicine, National Taiwan University, Taipei, Taiwan

**Keywords:** Hsp90, co-chaperone, FKBP4, client, chaperonin, CCT8

## Abstract

Hsp70, Hsp90, and chaperonin complexes are three essential molecular chaperones facilitating protein folding within eukaryotic cells. However, the important interplay among these systems is incompletely understood. FKBP4 is a co-chaperone of Hsp90 and exhibits increased expression in multiple cancer types. In this study, we employed two proximity-dependent biotin identification (BioID) systems to explore potential clients of the FKBP4-Hsp90 complex. Analysis of BioID mass spectrometry data revealed that the top category of the FKBP4-associated protein is cadherin-binding proteins, and one of the cadherin-binding proteins is a subunit of the chaperonin containing TCP-1 complex, CCT8. Furthermore, knockdown of FKBP4 led to CCT8 aggregation and compromised the stability of its clients, CDK2 and α-tubulin, indicating the dependency of the FKBP4-Hsp90 complex on CCT8 folding. These findings suggest that CCT8 is a client of the FKBP4-Hsp90 complex, implying a functional crosstalk between two of the three protein folding systems in eukaryotic cells.

Proteostasis encompasses the cellular processes that maintain the proper concentration, conformation, and functionality of proteins. This is achieved through a complex proteostasis network, composed of molecular chaperones and proteolytic pathways, that accurately maintains the folding and degradation of proteins. Molecular chaperones play critical roles in facilitating protein folding and preventing misfolding or aggregation. The principal folding systems in eukaryotic cells include Hsp70, Hsp90, and chaperonins, each fulfilling distinct but complementary functions (1). Hsp70 acts at an early stage by binding to nascent or unfolded polypeptides, preventing aggregation, and assisting in the initial folding steps through ATP-dependent cycles, with the aid of co-chaperones that enhance substrate specificity and facilitate release. Hsp90 functions either independently or downstream of Hsp70 by accepting partially folded client proteins for final maturation, a process often coordinated through their assembly into a Hop-mediated multichaperone complex that facilitates efficient protein folding (1, 2, 3). Chaperonins, characterized by their ring-shaped multi-subunit architecture, provide a protective cylindrical chamber, where specific polypeptides fold in isolation (4). The interplay among these systems is exemplified by the Hsp70-Hsp90 chaperone cascade, wherein Hsp70 first stabilizes client proteins before transferring them to Hsp90 for further folding and maturation (1, 2). Another example occurs during co-translational folding, where Hsp70 binds early to domain-specific sites, while TRiC chaperonin engages later with exposed hydrophobic regions of nearly folded domains (5). These coordinated cooperations ensure efficient protein folding, quality control, and, when necessary, the targeting of aberrant proteins for degradation, thereby maintaining cellular proteostasis.

The Hsp90 chaperone cycle is closely regulated by a set of proteins known as co-chaperones. A previous study demonstrates that the yeast Hsp90 co-chaperone Ids2 stimulates the chaperone activity of Hsp90 under calorie restriction, facilitating cells to maintain protein quality for prolonging longevity (6). *IDS2* deletion causes mitochondrial deficiency, leading to aging in yeast (7). Furthermore, sequence alignments reveal FK506-binding protein 4 (FKBP4) as a homolog of Ids2 in humans (6). FKBP4, also known as FKBP52, belongs to the family of immunophilins, which structurally contain peptidyl-prolyl isomerase (PPIase) domains (8). The PPIase domains are located at the N-terminal region of FKBP4 and comprise FKBP12-like domains 1 and 2 (FK1 and FK2). Specifically, the FK1 domain contains PPIase enzymatic activity, susceptible to inhibition by FK506 and rapamycin, whereas the FK2 domain primarily serves a structural function. The tetratricopeptide repeat (TPR) domain, located at the C-terminal end of FKBP4, possesses the capacity to engage in protein-protein interactions (9) and form complexes with Hsp90 (10).

As an Hsp90 co-chaperone, FKBP4 modulates the folding and activity of steroid receptors, including glucocorticoid, mineralocorticoid, androgen, progesterone, and estrogen receptor (11, 12, 13, 14). This holds particular significance in the context of hormone-dependent breast cancer (15), prostate cancer (16), and non-small-cell lung cancer progression (17). FKBP4 controls the activity of other clients, such as tau, which is a key pathological cause in several human neurodegenerative diseases (9), especially Alzheimer's disease. Moreover, FKBP4 may interact with the HSP70/RelA complexes to promote NF-κB nuclear translocation (18).

Here, we conducted BioID and mass spectrometry analyses to look for the interactome of FKBP4. The screen identified many cadherin-binding proteins, including CCT8. CCT8 is a chaperonin subunit containing tailless complex polypeptide 1, also known as CCT, TRiC, TCP-1, or TCP-1-ring complex. As a member of the chaperonin family, CCT functions in protein folding within the cytosol (19). Interestingly, knockdown of FKBP4 resulted in CCT8 aggregation and altered the folding of other CCT subunits. In addition, knockdown of FKBP4 impaired the folding function of CCT, causing the destabilization of CDK2 and α-tubulin. These findings discover a crosstalk between two protein folding systems in eukaryotic cells.

## Results

### BioID assay for screening FKBP4 interactome

The BioID technique relies on proximity-dependent biotinylation mediated by a bacterial biotin ligase (*E. coli* BirA R118G), which retains a conserved catalytic domain that binds biotin and ATP to produce biotinyl-5′-AMP. The R118G mutation increases the biotin dissociation constant by 100-fold and the biotinyl-5′-AMP dissociation rate by 400-fold, thereby facilitating the labeling of nearby proteins (20, 21). Therefore, the advantage of the BioID method is that it can isolate transiently interacting proteins within their normal cellular context. A major limitation of fusion protein-based approaches is the potential interference with accurate subcellular localization. To address this, *Aquifex aeolicus* BirA was similarly mutated to generate BioID2, a smaller (27 kDa) promiscuous biotin ligase. Compared to the larger BioID (35 kDa), which has been shown to impair the proper targeting of certain fusion proteins, BioID2 reduces steric hindrance and exhibits enhanced efficiency in proximity-dependent biotinylation. Notably, BioID2 also demonstrates an expanded biotinylation range and increased labeling efficacy, enabling the detection of proteins that are otherwise refractory to labeling by the BioID (21). To explore the downstream clients of FKBP4 as well as possible, both BioID and BioID2 proximity-labeling approaches were employed to screen for the interactome of FKBP4 in HEK293T cells.

**Figure 1:**
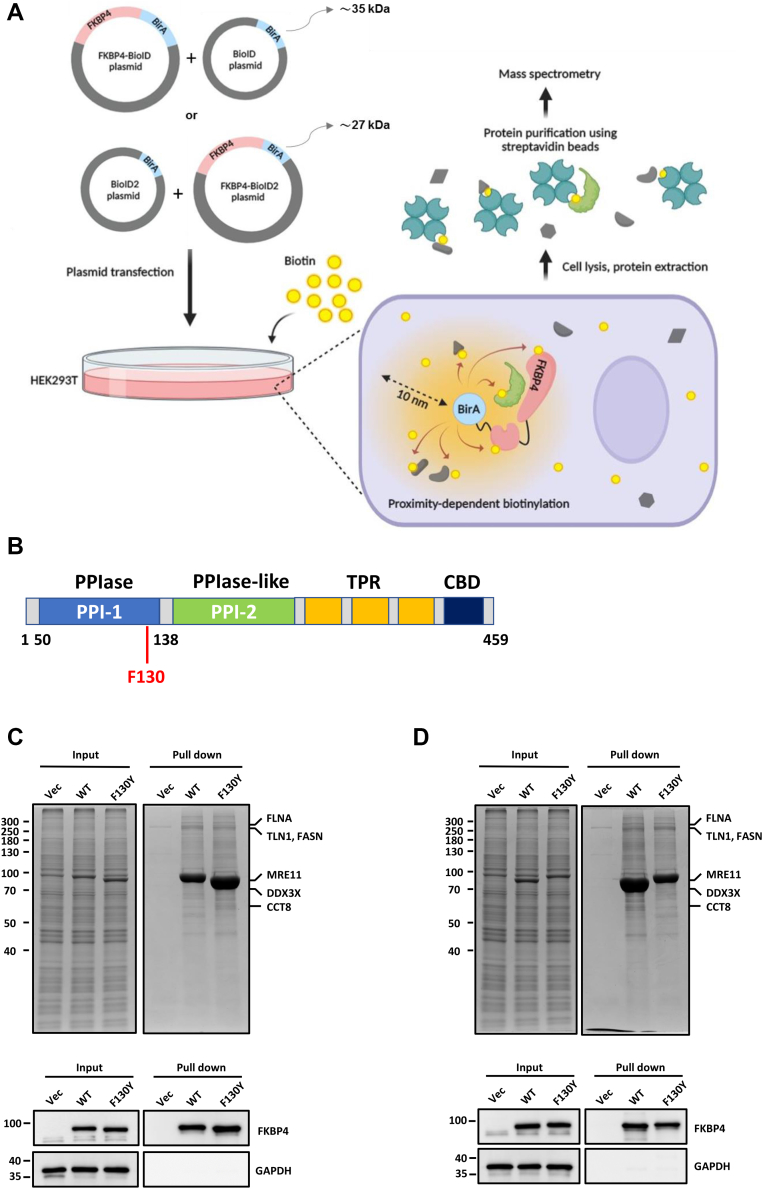
**Bio-ID assay is employed to capture biotin-labeled FKBP4-interacting proteins.***A*, the schematic illustrating the BioID assay protocol. The BioID plasmid contains the gene for biotin ligase, BirA. The FKBP4 gene is integrated into the BioID plasmid, which is then transfected into HEK293T cells along with the BioID plasmid. After 24 h, biotin is added to the cell culture medium for another 24-h incubation. BirA is fused to FKBP4 to biotinylate interacting proteins. Streptavidin beads capture biotinylated proteins, enabling mass spectrometry analysis to identify FKBP4's potential clients. *B*, the primary structure of FKBP4. FKBP4 comprises one FK1 domain (possessing PPIase activity), one FK2 domain (lacking PPIase activity), and three TPR domains (tetratricopeptide repeats). *C*, *upper panel*: HEK293T cells were transfected with plasmids encoding pcDNA3.1 MCS-BirA(R118G)-HA (Vec), pcDNA3.1 FKBP4-BirA(R118G)-HA (WT), or pcDNA3.1 FKBP4 F130Y-BirA (R118G)-HA (F130Y), followed by the execution of the BioID assay. Proteins biotinylated by BirA were captured using streptavidin beads, separated *via* SDS-PAGE, and stained by Coomassie brilliant blue. To minimize the potential overlap between FKBP4 and proteins of interest with similar molecular weights, each lane was segmented into three regions based on the migration position of FKBP4. The vector (Vec) control lanes were divided into two regions - above and below the FKBP4 reference position. Protein labeling was guided by predicted molecular weights of the candidate interactors. The *lower panel* is the western blotting to confirm the overexpression of FKBP4 using the indicated antibodies. *D*, HEK293T cells were transfected with plasmids encoding pcDNA3.1 MCS-BioID2-HA (Vec), pcDNA3.1 FKBP4-BioID2-HA (WT), or pcDNA3.1 FKBP4 F130Y-BioID2-HA (F130Y). The experimental procedure for the *upper* and *lower panels* mirrored that of (*C*).

Since enzymes are utilized to speed up chemical reactions, we speculate that enzyme-dead bait may retain interacting proteins longer than the wild-type (WT) one. Sequence alignment and functional analysis demonstrated that phenylalanine 130 of FKBP4 is located at the active site of PPIase, and the replacement of phenylalanine 130 with tyrosine (F130Y) impairs the PPIase activity of FKBP4 (22, 23). We therefore first generated WT and the PPIase activity-dead mutant of FKBP4 to conduct the BioID pull-down assay using both BioID and BioID2 systems (21, 24). After obtaining pull-down samples, we subjected them to SDS-polyacrylamide gel electrophoresis (SDS-PAGE), and the gels were stained with Coomassie brilliant blue to check whether streptavidin beads could pull down FKBP4 and its potential clients. Compared to the vector controls, FKBP4 and FKBP4 F130Y samples pulled down more bands in both BioID and BioID2 systems (Fig. 1, *C* and *D* top panel), confirming the capture of interacting proteins. Concurrently, Western blot analysis confirmed the successful overexpression of FKBP4 (Fig. 1, *C* and *D* bottom panel). The streptavidin bead-bound protein samples were reconstituted in a buffer and analyzed *via* LC-MS/MS analysis for quantitative proteomics to determine the FKBP4-interacting proteins.

### LC-MS/MS analysis identifies FKBP4 interacting proteins

We collected a total of 86,368 MS/MS spectra from six samples. The complete FKBP4-interacting proteins in HEK293T cells are listed in Files S1–S4. We initially categorized the proteins into four groups: segregating first-generation and second-generation BioID proteomes, and further classifying them based on whether the scores of the F130Y mutant of FKBP4 were greater than those of the FKBP4 WT and whether the scores of the FKBP4 WT were greater than those of the vector control. The rationale for selecting FKBP4 F130Y > FKBP4 WT was based on the assumption that the PPIase-dead mutation may cause FKBP4 clients to halt at the intermediate of the FKBP4-mediated folding process, which enables clients to be trapped on FKBP4. The reason for choosing FKBP4 WT > vector score was to avoid missing any other proteins that could potentially be FKBP4 clients.

**Figure 2:**
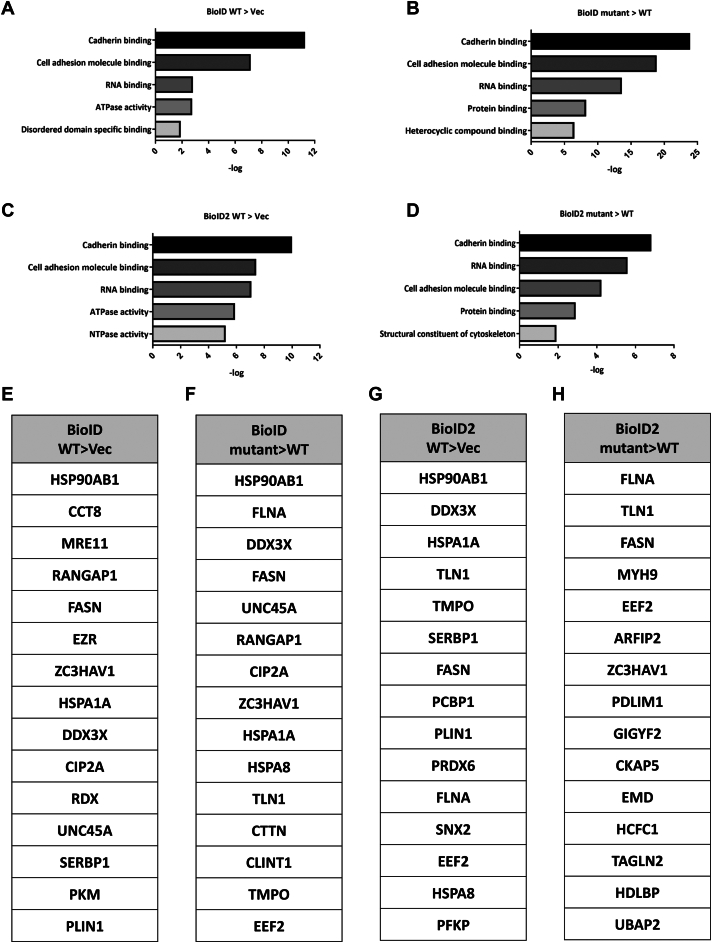
**The most significantly enriched GO terms and the top 15 proteins in the category of cadherin-binding proteins in the FKBP4 interactome.***A*, GO analysis of the FKBP4-interacting proteins showed an increased binding in cells overexpressing FKBP4 WT-BirA-HA than in cells overexpressing the vector. *B*, GO analysis of the FKBP4-interacting proteins showed an increased binding in cells overexpressing FKBP4 F130Y-BirA-HA than in cells overexpressing FKBP4 WT-BirA-HA. *C*, GO analysis of the FKBP4-interacting proteins showed an increase in binding in cells overexpressing FKBP4 WT-BioID2-HA than in cells overexpressing the vector. *D*, GO analysis of the FKBP4-interacting proteins showed an increased binding in cells overexpressing FKBP4-BioID2-HA F130Y than in cells overexpressing FKBP4 WT-BioID2-HA. *E*, the top 15 proteins in the cadherin-binding category of *panel* (*A*). *F*, the top 15 proteins in the cadherin-binding category of *panel* (*B*). *G*, the top 15 proteins in the cadherin-binding category of *panel* (*C*). *H*, the top 15 proteins in the cadherin-binding category of *panel* (*D*).

We first utilized the Gene Ontology (GO) Resource website to analyze the results. Surprisingly, in the four groups, the most significant protein category was the cadherin-binding proteins (Fig. 2, *A*–*D* and Files S5–S8). Among these cadherin-binding proteins, we arranged them by scoring them from highest to lowest. The top 15 cadherin-binding proteins recovered from each group are listed in Figure 2, *E*–*H*. We selected at least two proteins with the highest scores from each category, excluding heat shock proteins, for further study. FLNA and DDX3X were initially selected based on their high enrichment rankings, excluding Hsp90, in the BioID FKBP4 mutant > WT group (Fig. 2*F*). Subsequently, TALIN-1 and FASN were chosen due to their relatively high ranking among the remaining candidates, excluding FLNA (Fig. 2*H*). As mentioned above, to achieve a comprehensive identification of FKBP4 clients, the BioID and BioID2 FKBP4 WT > vector control groups were analyzed. Following the exclusion of overlapping proteins across functional categories, CCT8 and MRE11 were selected based on their notable enrichment (Fig. 2, *E* and *G*). We, therefore, focused on a total of six potential clients, FLNA, DDX3X, TALIN-1, FASN, CCT8, and MRE11, for further examination. Filamin-A (FLNA) is an actin-binding protein that regulates cytoskeletal rearrangement by interacting with integrins, transmembrane receptors, and second messengers (25). It also plays a role in calcium signaling by forming protein complexes with calcium-sensing receptors Trio and Rho, facilitating E-cadherin-mediated cell–cell adhesion (26). p53 inactivation causes the reduction of DDX3X, contributing to cancer progression through the MDM2/Slug/E-cadherin pathway. This process entails decreased MDM2 level, which upregulates Slug expression, further inhibiting E-cadherin and promoting metastasis in non-small-cell lung cancers (27). The expression level of Talin-1 is reduced in endometrial cancer tissue (28), and Talin-1 promotes prostate cancer metastasis (29). A previous study indicates an interaction between TALIN-1 and E-cadherin (30). A high level of fatty acid synthase (FASN) is found in breast cancer (31). FASN also contributes to epithelial-mesenchymal transition (EMT) by regulating the expression of E-cadherin and N-cadherin in ovarian cancer (32). CCT8 plays a key role in various cancers such as non-Hodgkin's lymphoma (33), glioma (34), colon cancer, and hepatocellular carcinoma (35). CCT8 primarily functions in protein folding within the cytosol and interacts with E-cadherin (30). MRE11 plays a crucial role in repairing DNA double-strand breaks *via* homologous recombination (36). Moreover, MRE11 is also known to interact with E-cadherin (30). Overall, these six potential clients are integral to E-cadherin's functionality and related pathways.

### Knockdown of FKBP4 reduces E-cadherin protein level

**Figure 3:**
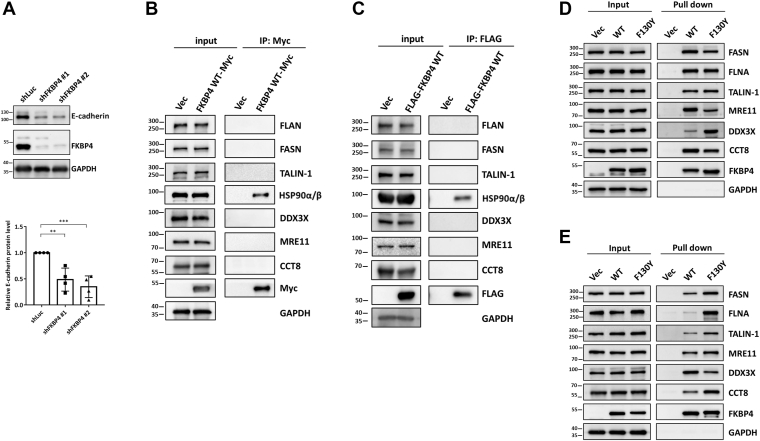
**Validation of the findings from mass spectrometry and GO term analyses.***A*, the upper panel: Detection of E-cadherin protein levels within HCT116 cells following FKBP4 knockdown using western blotting. The *lower panel*: Quantitative analysis of E-cadherin levels with GAPDH as an internal control. Data was analyzed with an unpaired two-tailed Student's *t* test. Each dataset is expressed as mean ± SD for n = 3. ∗∗*p* < 0.01, ∗∗∗*p* < 0.001. *B*, after 48 h of transfection, vector or Myc-tagged FKBP4 WT-expressing HEK293T cell lysates were precipitated by anti-c-Myc antibodies, and the precipitants were detected for the co-purification of the endogenous proteins. *C*, after 48 h of transfection, vector, FLAG-tagged FKBP4 or FLAG-tagged FKBP5 expressing HEK293 T cell lysates were precipitated by anti-FLAG antibodies, and the products were detected for the co-precipitation of the endogenous proteins. *D*, BioID assay was performed in HEK293T cells overexpressing BirA (R118G)-HA (Vec), FKBP4-BirA (R118G)-HA (WT), or FKBP4 F130Y-BirA (R118G)-HA (F130Y). Biotinylated proteins were captured using streptavidin beads, and western blotting was employed to detect interactions between six candidates and FKBP4. *E*, similar experimental procedures to (*C*) were conducted in HEK293T cells overexpressing BioID2-HA (Vec), FKBP4-BioID2-HA (WT), or FKBP4 F130Y-BioID2-HA (F130Y).

According to the results of GO term analysis (Fig. 2), the most significant protein category in all 4 groups is the cadherin-binding proteins. Given that TALIN-1, MRE11, and CCT8 interact with E-cadherin (30), that FASN, FLNA, and DDX3X are implicated in EMT (27, 32, 37, 38), and that FKBP4 expression is upregulated in colon cancers (39), we examined E-cadherin expression in human colorectal carcinoma HCT116 cells under the FKBP4 knockdown. Interestingly, a significant decrease in E-cadherin level was observed in both shFKBP4 knockdown strains (Fig. 3*A*), suggesting that FKBP4 controls E-cadherin level.

Although the interacting proteins were identified in mass spectrometry analysis, to confirm their interactions with FKBP4, we first employed the co-immunoprecipitation (Co-IP) analysis to investigate the interaction between FKBP4 and these six candidates. However, the interaction signal between these six candidates and FKBP4 could not be detected by Co-IP analysis (Fig. 3, *B* and *C*). We next investigated the potential interaction between FKBP4 and selected candidate proteins using the yeast two-hybrid assay. Three of the candidates were relatively large (FLNA, 280 kDa; TALIN-1, 270 kDa; FASN, 270 kDa), which posed challenges for their expression in yeast cells. Hence, we focused on expressing CCT8, DDX3X, and MRE11 as LexA fusion proteins to assess their interaction with FKBP4 fused to the transcriptional activation domain, as indicated by the *LEU2* reporter gene expression in the yeast two-hybrid system (40). Expression of FKBP4 alone (as prey) and MRE11 alone (as bait) resulted in low and high levels of leaky *LEU2* expression, respectively (Fig. S1). None of the constructs of CCT8, DDX3X, or MRE11 showed *LEU2* reporter activity above the background observed in the negative controls, suggesting a lack of detectable interaction under these assay conditions.

Next, we conducted the BioID system utilizing BioID plasmid-transfected and biotin-cultured cells, and bead-bound protein extracts were subjected to gel electrophoresis, followed by immunoblotting using specific antibodies against each candidate (Fig. 3, *D* and *E*). The results exhibited that all six candidates were pulled down in the pellets of both BioID systems (Fig. 3, *D* and *E*), indicating that FASN, FLNA, TALIN-1, MRE11, DDX3X, and CCT8 are all FKBP4-interacting proteins. Therefore, these results highlight that the BioID system exhibits greater sensitivity as a detection approach compared to the Co-IP and yeast two-hybrid analyses.

### FKBP4 regulates CCT8's functionality by preventing its aggregation

**Figure 4:**
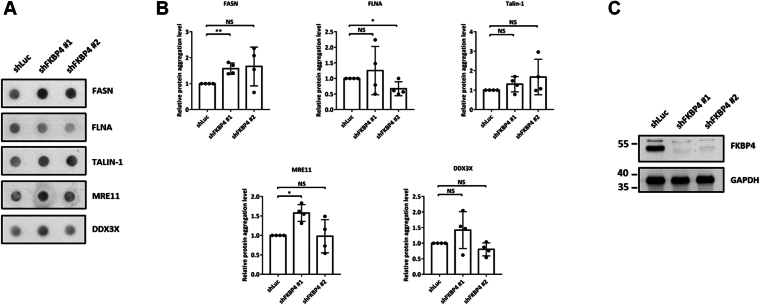
**Loss of FKBP4 does not induce the protein aggregation of FASN, FLNA, TALIN-1, MRE11, and DDX3X.***A*, following the knockdown of FKBP4 in HCT116 cells for 72 h, the aggregation of clients within cells was assessed using a filter trap assay. Cell lysates were filtered through a nitrocellulose membrane, and the retained proteins on the membrane were detected using specific antibodies against FASN, FLNA, TALIN-1, MRE11, and DDX3X. *B*, quantitative analysis of the aggregation of FASN, FLNA, TALIN-1, MRE11, and DDX3X, with GAPDH serving as an internal control. All data were analyzed with an unpaired two-tailed Student's *t* test. *C*, before the filter trap assay was conducted, Western blot analysis was used to assess the knockdown efficiency of FKBP4. Chemiluminescence images of FKBP4 and GAPDH are presented. Each dataset is expressed as mean ± SD for n = 3.

**Figure 5:**
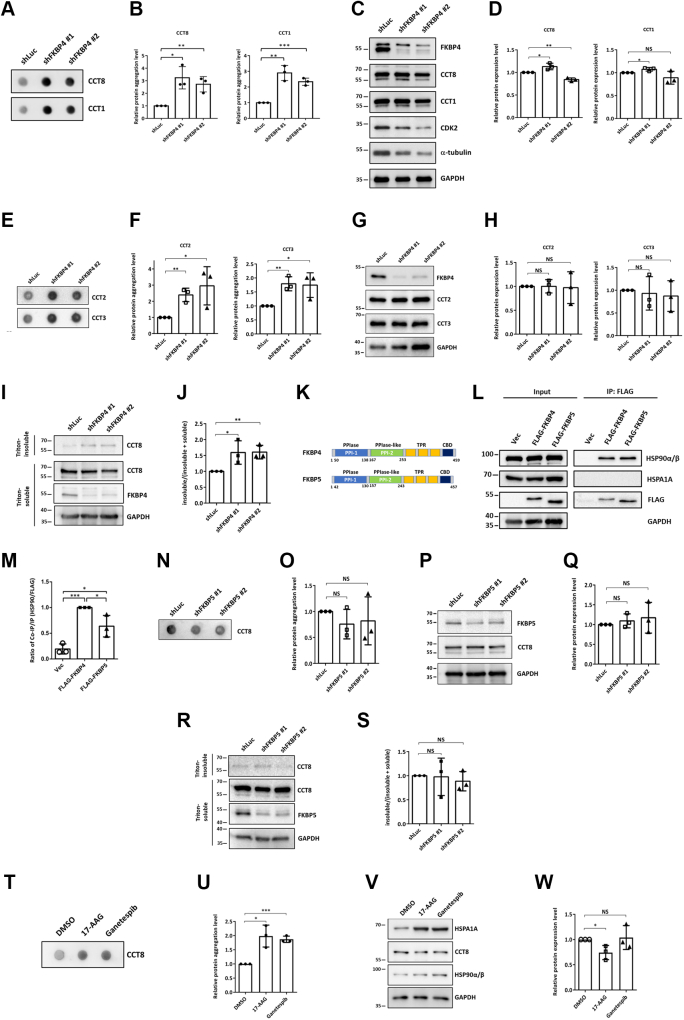
**FKBP4 prevents CCT8 aggregation.***A*, following the knockdown of FKBP4 in HCT116 cells for 72 h, the aggregation of CCT1 and CCT8 proteins within cells was assessed using a filter trap assay. Cell lysates were filtered through a nitrocellulose membrane, and the retained proteins on the membrane were detected using specific antibodies against CCT1 and CCT8. *B*, quantitative analysis of CCT1 and CCT8 protein aggregation, normalized to GAPDH, in shFKBP4 HCT116 cells. *C*, cell lysates that did not pass through the nitrocellulose membrane were subjected to western blotting to detect the levels of FKBP4, CCT8, CCT1, CDK2, α-tubulin, and GAPDH within the cells. *D*, quantitative analysis of CCT8 and CCT1 protein expression, normalized to GAPDH, in shFKBP4 HCT116 cells. *E*, following the knockdown of FKBP4 in HCT116 cells for 72 h, the aggregation of CCT2 and CCT3 proteins within cells was assessed using a filter trap assay. The experimental procedure mirrored that of (*A*). *F*, quantitative analysis of CCT2 and CCT3 protein aggregation, normalized to GAPDH, in shFKBP4 HCT116 cells. *G*, cell lysates that did not pass through the nitrocellulose membrane were subjected to western blotting to detect the levels of FKBP4, CCT2, CCT3, and GAPDH within the cells. The experimental procedure mirrored that of (*C*). *H*, quantitative analysis of CCT2 and CCT3 protein expression, normalized to GAPDH, in shFKBP4 HCT116 cells. *I*, shFKBP4 HCT116 cells were harvested after 3 days of puromycin selection. The cell lysates were biochemically fractionated into Triton-soluble and -insoluble fractions as described in “Experimental procedures”. The FKBP4 expression levels and Triton-soluble and -insoluble fractions of CCT8 were analyzed by western blotting. The GAPDH was used as a loading control. *J*, The ratio of the CCT8 Triton-insoluble form in (*I*). *K*, The primary structures of FKBP4 and FKBP5. *L*, after 48 h of transfection, vector, FLAG-tagged FKBP4 or FLAG-tagged FKBP5 expressing HEK293T cell lysates were precipitated by anti-FLAG antibodies, and the products were detected for the co-purification of the endogenous proteins. *M*, comparison of the interaction between Hsp90 and FLAG-tagged FKBP4 or FLAG-tagged FKBP5 in (*L*). *N*, following the knockdown of FKBP5 in HCT116 cells for 72 h, the aggregation of CCT8 proteins within cells was assessed using a filter trap assay. The experimental procedure mirrored that of (*A*). *O*, quantitative analysis of the protein aggregation of CCT8, normalized to GAPDH, in shFKBP5 HCT116 cells. *P*, cell lysates that did not pass through the nitrocellulose membrane were subjected to western blotting to detect the levels of FKBP5, CCT8, and GAPDH within the cells. The experimental procedure mirrored that of (*C*). *Q*, quantitative analysis of CCT8 protein expression, normalized to GAPDH, in shFKBP5 HCT116 cells. *R*, shFKBP5 HCT116 cells were harvested after puromycin selection for 3 days. The experimental procedure mirrored that of (*I*). *S*, the ratio of the CCT8 Triton-insoluble form in (*R*). *T*, HCT116 cells were treated with 17-AAG (20 μM) or ganetespib (2 μM) for 24 h, and CCT8 aggregation was assessed by filter trap assay. *U*, quantitative analysis of CCT8 protein aggregation with GAPDH serving as an internal control in (*T*). *V*, cell lysates that did not pass through the nitrocellulose membrane were subjected to western blotting to detect the levels of HSPA1A, CCT8, HSP90, and GAPDH within the cells. The experimental procedure mirrored that of (*C*). *W*, quantitative analysis of CCT8 protein expression was normalized with GAPDH in (*V*). All data were analyzed with an unpaired two-tailed Student's *t* test. Each dataset is expressed as mean ± SD for n = 3. ∗*p* < 0.05, ∗∗*p* < 0.01, ∗∗∗*p* < 0.001.

Since chaperones facilitate protein folding and some unfolded proteins tend to aggregate, we speculated that, in the absence of the Hsp90 co-chaperone FKBP4, some FKBP4 clients may be unfolded and subjected to aggregation. To investigate whether the six candidate proteins (FLNA, DDX3X, TALIN-1, FASN, CCT8, and MRE11) exhibit intracellular aggregation in the absence of FKBP4, FKBP4 expression was repressed in HCT116 cells using lentiviral-mediated knockdown. Aggregation of these proteins was subsequently assessed *via* filter trap assay. Notably, FASN, FLNA, TALIN-1, MRE11, and DDX3X displayed no significant changes in aggregation following FKBP4 depletion (Fig. 4, *A* and *B*). Western blot analysis was used to verify the knockdown efficiency of FKBP4 in HCT116 cells (Fig. 4*C*). In contrast, only CCT8, a member of the chaperonin family, exhibited pronounced aggregation in FKBP4-deficient cells (Fig. 5, *A*–*C*), suggesting that loss of FKBP4 leads to the accumulation of unfolded CCT8 in an aggregated state. Considering that the TTC complex comprises a multi-protein ring-shaped complex composed of eight subunits, we sought to investigate whether knockdown of FKBP4 and the consequent aggregation of CCT8 protein would influence the folding of other TTC subunits. Utilizing CCT1 as a representative, the filter trap assay demonstrated aggregation of CCT1 in the FKBP4-knockdown HCT116 cells (Fig. 5, *A* and *B*). Western blot analysis demonstrated that the expression of CCT8 and CCT1 proteins is not influenced by the FKBP4 knockdown (Fig. 5, *C* and *D*). These findings suggest that FKBP4 knockdown induces aggregation of the CCT8 and may further impair the folding of other chaperonin subunits. To validate this hypothesis, a filter trap assay was further performed to determine whether knockdown of FKBP4 induces aggregation of CCT2 and CCT3. The results indicated that FKBP4 knockdown promotes the aggregation of CCT2 and CCT3 as well in HCT116 cells (Fig. 5, *E*–*G*). Notably, the overall protein expression levels of CCT2 and CCT3 remained largely unchanged in FKBP4-deficient HCT116 cells (Fig. 5, *G* and *H*). These results indicate that the folding of other CCT subunits may also be altered under the FKBP4 knockdown.

Following the observed CCT8 aggregation in FKBP4 knockdown cells (Fig. 5, *A* and *B*), we next investigated the functional consequences of CCT8 perturbation. A previous study demonstrated that knockdown of CCT8 inhibits cell proliferation and blocks entry into the S phase (41). The study also revealed that, under the CCT8 knockdown, CDK2 exhibits a decreased expression level. Therefore, we conducted FKBP4 knockdown in HCT116 cell lines and assessed the protein expression levels using western blotting. The results showed that FKBP4 knockdown decreased CDK2 expression (Fig. 5*C*). Additionally, several studies suggest that cytoskeletal proteins, particularly tubulin, are the primary clients of CCT (42, 43, 44). *In vivo*, α-tubulin monomers undergo folding through a folding pathway comprising prefoldin and CCT (45). Hence, we hypothesize that α-tubulin, when not folded by CCT, may display decreased cellular abundance due to its instability. Knockdown of FKBP4 indeed reduced the stability of α-tubulin (Fig. 5*C*). These findings imply that knockdown of FKBP4 leads to the aggregation of CCT8 protein, and the defective CCT function may subsequently result in an increased level of unfolded α-tubulin in cells. To further confirm that CCT8 aggregation is induced by FKBP4 knockdown, we performed cell fractionation analysis to examine the distribution of CCT8 in Triton X-100-soluble and -insoluble fractions. The results demonstrated that FKBP4 knockdown leads to an increased proportion of CCT8 in the insoluble fraction (Fig. 5, *I* and *J*). FKBP5 displays 77% similarity to FKBP4 at the amino acid level (11, 46) (Figs. 5*K* and S2) and is also involved in steroid hormone receptor signaling. Through the Co-IP analysis, we found that FKBP5 exhibits a lower binding affinity to Hsp90 compared to FKBP4 (Fig. 5, *L* and *M*). FKBP4 did not co-precipitate HSP70 in HEK293T cells (Fig. 5*L*), unlike what was previously observed in lung cancer and bronchial epithelial cell lines (18). Given that both FKBP4 and FKBP5 are co-chaperones of Hsp90 (Fig. 5, *L* and *M*), we examined whether FKBP5 knockdown can also cause the aggregation of CCT8. The results of the filter trap assay indicated that knockdown of FKBP5 cannot induce CCT8 protein aggregation (Fig. 5, *N* and *O*) and affect CCT8 expression level (Fig. 5, *P* and *Q*). In addition, cell fractionation analysis demonstrated that knockdown of FKBP5 cannot induce the formation of insoluble CCT8 (Fig. 5, *R* and *S*). These results suggested the specificity between FKBP4 and CCT8. We also used Hsp90 inhibitors (17-AAG and ganetespib) to examine whether they can induce CCT8 aggregation. Consistent with previous studies, HSPA1A expression was increased upon treatment (47, 48) (Fig. 5*V*), and filter trap assays revealed an increase in CCT8 aggregation (Fig. 5, *T* and *U*). Importantly, CCT8 protein expression levels were not altered in HCT116 cells treated with Hsp90 inhibitors (Fig. 5, *V* and *W*). Together, these findings suggest a specific regulatory pathway between Hsp90-FKBP4 and CCT8.

## Discussion

In this study, we established a new approach to screen for potential clients of the FKBP4 co-chaperone. This method operates on a principle like immunoprecipitation, with the distinction lying in the fact that, in immunoprecipitation, proteins must maintain continuous physical interaction with the target proteins until they are eventually bound by antibodies and beads. In contrast, the BioID system allows for the attachment of biotin to potential clients during the cell culture stage. Consequently, even transient interactions can be identified, significantly enhancing the sensitivity of capturing potential clients of chaperones and co-chaperones. In the future, this highly sensitive approach can be used for the client screening of different chaperones and co-chaperones.

Hsp90 functions as a homodimeric complex composed of an N-terminal nucleotide-binding domain (NBD), a middle domain (M), and a C-terminal dimerization domain (CTD) (49). Co-chaperones interact with distinct domains of Hsp90 to stabilize its conformation and modulate its activity. For instance, the N-terminal region of the co-chaperone Aha1 binds the M domain of Hsp90 to enhance its ATPase activity (50). In the previous study, we discovered that S148 phosphorylation of Ids2 impedes the ATPase activity of the yeast Hsp90 (6). Domain-mapping analyses further revealed that Ids2 binds the M domain of Hsp90 and promotes its ATPase activity (6). These findings imply that Ids2 functions not only as a regulator but also as a co-chaperone of Hsp90. In this study, we identified FKBP4 as a co-chaperone of Hsp90 capable of regulating chaperonin folding. Given the homology between FKBP4 and Ids2 (6) and the co-chaperone role of FKBP4, we hypothesize that Ids2 may serve as the co-chaperone and similarly influence chaperonin folding. Future investigations into the functional relationship between Ids2 and chaperonins in yeast will provide further insights into this potential regulatory mechanism.

We notice that the FKBP4 F130Y mutant shows faster migration than the WT protein in the BioID system but migrates more slowly than the WT in the BioID2 system. This difference may result from altered post-translational modification induced by the F130Y mutation. This possibility is supported by previous Coomassie blue staining data, which show that altered post-translational modifications occur at the F130Y mutation in FKBP5, a protein structurally similar to FKBP4, within the conserved PPIase domain (23). Notably, FKBP4 and FKBP5 share a high degree of structural conservation, particularly within the PPIase domain. Importantly, the backbone regions within the BioID and BioID2 systems should not cause the observed molecular weight differences. However, different FKBP4 F130Y fusion proteins in two systems may display different structures. Given the structural differences between the BirA enzymes in BioID and BioID2, we propose that the post-translational modifications induced by the F130Y mutation may be differentially influenced by the fusion parts in the BioID and BioID2 systems.

In this study, BioID and BioID2 were employed to comprehensively identify potential FKBP4 client proteins. As previously described, structural differences between BioID and BioID2 influence their labeling efficiency, which likely contributes to the distinct interaction profiles. Excluding heat shock proteins, FLNA, DDX3X, FASN, and TALIN-1 rank among the top 15 interactors in Figure 2*F*, but FLNA, TALIN-1, and FASN exhibit improved rankings in Figure 2*H*. Although DDX3X is absent from Figure 2*H*, its presence in Figure 2, *F* and *G* suggests it remains a strong candidate as an FKBP4 interactor. Following the same rationale, CCT8 and MRE11 were selected as the top candidates from Figure 2*E*. Additionally, the observed differences in candidate profiles across Figure 2, *E*–*H* may reflect inherent specificity between BioID and BioID2 systems. Moreover, the yeast two-hybrid findings are consistent with the previous report, indicating that protein folding processes mediated by Hsp90 co-chaperones, such as FKBP4, are often transient and may not be readily captured by the yeast two-hybrid system (51), and detection of co-chaperone interactions by the yeast two-hybrid system typically requires stabilization of Hsp90 in its client-bound conformation, which can be achieved through mutations in the ATPase domain (51).

Here, we identified many new FKBP4 clients, including CCT8. When CCT8 loses its folding, other subunits of CCT may become unstable. Related research demonstrated previously that knockdown of CCT2 leads to the degradation of other endogenous CCT subunits to different extents (52). Similarly, our study showed that not only CCT8 but also CCT1, CCT2, and CCT3 aggregate in the FKBP4 knockdown cells. Therefore, we speculate that there may exist a mechanism among different CCT subunits whereby they mutually stabilize each other within the CCT complex. Moreover, the CCT/TRiC expression levels are usually higher in cancer cells than in normal cells (53). Elevated expression of CCT7 serves as a diagnostic biomarker for hepatocellular carcinoma (54). Increased expression of CCT2 in tumors obtained from patients with lung cancer is likewise correlated with diminished survival rates (55). As a member of the chaperonin family, CCT, especially CCT8, is necessary for the correct folding of many proteins. Processes such as signal transduction, the creation and reorganization of the cytoskeleton, the development of immune synapses, macromolecular conveyance, and cell division all rely on the functionality of the CCT complex (56, 57). Moreover, CCT8 inhibits the entry of p53 into the nucleus and promotes the cell cycle and metastasis in colorectal cancers (CRC) (58). Together with our results, we postulate that enhancing the folding of CCT8 may potentially stabilize and therefore boost the functionality of other CCT subunits, which provides a novel avenue for cancer therapy.

CRC is a prevalent and life-threatening malignancy, ranking as the third most commonly diagnosed cancer globally, following lung and breast cancers (59). Patient prognosis is closely linked to the tumor stage at the time of diagnosis, with early-stage detection significantly improving treatment outcomes. One hallmark of carcinoma progression is phenotypic plasticity, exemplified by EMT and mesenchymal-epithelial transition (MET). The change in epithelial integrity facilitates metastasis. A key molecular indicator of EMT and MET is the alteration of E-cadherin, a cell adhesion molecule, whose diminished expression reflects the loss of epithelial characteristics and acquisition of mesenchymal traits (60, 61). As we observed, the knockdown of FKBP4 leads to a reduced E-cadherin level in HCT116 cells. These findings may imply that FKBP4's function is critical for CRC metastasis.

In addition, numerous studies have demonstrated that knockdown of CCT8 suppresses cell proliferation by inhibiting kinases and cyclins associated with cell cycle progression (41, 58). While previous studies have predominantly focused on total CCT8 expression levels, our findings provide a novel perspective on CCT8 folding. Upon the knockdown of FKBP4, the aggregation of CCT8 is markedly increased. Notably, the accumulation of aggregated CCT8 is accompanied by a blockade of cell cycle progression. These observations suggest that reduced total CCT8 expression and an increase in non-functional, aggregated CCT8 proteins may contribute to the inhibition of cell proliferation.

**Figure 6:**
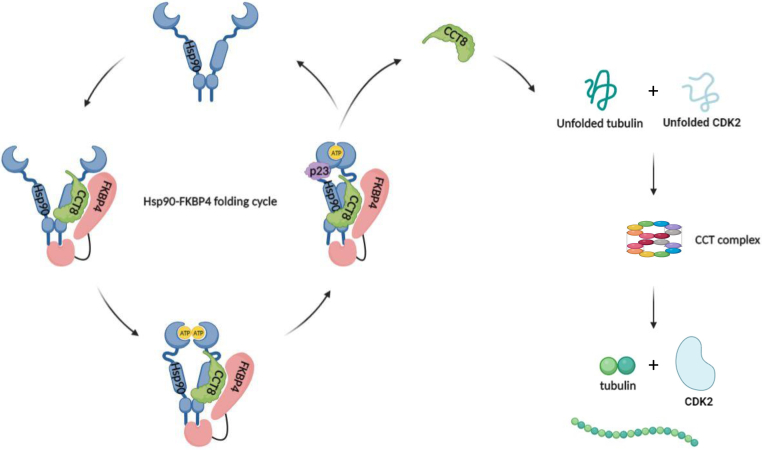
**The model of the Hsp90-FKBP4 folding cycle.** FKBP4 binds to Hsp90 through the C-terminal TRP motif, forming an Hsp90-FKBP4 protein complex to execute protein folding. FKBP4 can help fold CCT8, one of the TRiC subunits. CCT8, when well folded by the Hsp90-FKBP4 complex, can form a tubular chaperonin structure with other TRiC subunits. Functional chaperonin is known to be responsible for the folding of α-tubulin.

Hsp70, Hsp90, and chaperonin complexes play crucial roles as molecular chaperones, facilitating protein folding within eukaryotic cells. A significant portion of clients may transition from Hsp70 to Hsp90 during activation processes. Among these clients, the glucocorticoid receptor (GR) stands out as one regulated by both Hsp70 and Hsp90. Several studies have highlighted the involvement of various chaperones and co-chaperones, such as J-domain protein, Hsp70, Hop, p23, and Hsp90, in the activation of GR (62). Specifically, Hsp70 aids in the unfolding of the hormone-binding region within the GR ligand-binding domain. The attainment of the hormone-binding state requires the sequential coordinated efforts of Hsp90 and its co-chaperones, Hop, and p23, for proper GR folding (63). This underscores the reciprocal interplay and contribution between Hsp70 and Hsp90 in the folding of certain clients. In this study, we found that the systems involving Hsp90 and chaperonin are not mutually exclusive, as CCT subunits aggregate under the FKBP4 knockdown (Fig. 6). From our perspective, within the realm of specific client protein folding, a potential regulatory linkage may exist between the Hsp90-FKBP4 and chaperonin folding systems. Additionally, FKBP4 may associate with the HSP70/RelA complex to facilitate NF-κB nuclear translocation (18). Future studies will aim to generate an FKBP4 mutant that selectively disrupts the FKBP4-HSP70 interaction while preserving FKBP4-HSP90 binding, to determine whether this alteration also induces CCT8 aggregation and thereby clarify HSP70's role in FKBP4-mediated CCT8 folding.

## Experimental procedures

### Cell culture, transfection, and reagents

HEK293T and HCT116 cells were maintained in Dulbecco's Modified Eagle Medium (DMEM) containing 10% fetal bovine serum and antibiotics (100 U/ml penicillin and 100 μg/ml streptomycin) at 37 °C with 5% CO_2_. 293T cells were transfected with T-Pro Non-liposome Transfection Reagent (T-Pro Biotechnology) according to the manufacturer's instructions. HCT116 cells were treated with the Hsp90 inhibitors 17-AAG (MedChem Express) and ganetespib (MedChem Express) at final concentrations of 20 and 2 μM, respectively.

### Lentivirus production and infection

HEK293T cells were co-transfected with the packaging plasmid (pCMV-Δ8.91), envelope (pMD.G), and either hairpin pLKO-RNAi vectors (National RNAi Core Facility, Institute of Molecular Biology/Genomic Research Centre, Academia Sinica, Taiwan) for the virus production. After 24 h post-transfection, the medium was replaced with DMEM-high glucose medium containing filtered 1% BSA medium. Virus-containing supernatants were collected after 48 h and 72 h post-transfection. For a 60 mm × 60 mm dish culture, cells were infected with each virus plus DMEM-high glucose medium containing 0.8 μg/ml polybrene (Millipore) for 24 h. The transduced cells were selected with DMEM-high glucose medium containing 1 μg/ml puromycin (Sigma-Aldrich) for 3 days.

### Plasmids

The WT or mutant FKBP4 was cloned into the pGEM-T Easy vector (Promega, Madison, WI, USA). pcDNA3.1 MCS-BirA (R118G)-HA was acquired from Addgene #36047. The pTA-FKBP4 WT or pTA-FKBP4 F130Y was PCR-amplified and cloned into the NheI and AgeI sites of the pcDNA3.1 MCS-BirA (R118G)-HA. pcDNA3.1 MCS-BioID2-HA was purchased from Addgene #74224. The pTA-FKBP4 WT or pTA-FKBP4 F130Y was PCR-amplified and cloned into the NheI and AgeI sites of the pcDNA3.1 MCS-BioID2-HA. The pEG202-*CCT8*, pEG202-*MRE11*, pEG202-*DDX3X*, and pJG4-5-*FKBP4* were generated using In-Fusion HD Cloning Kits (Takara) at the BioMed Resource Core of the first Core Facility Lab, NTU-CM.

### Antibodies

Primary antibodies were used to detect FKBP4 (1:1000, ab97306, Abcam), GAPDH (1:1000, GTX100118, GeneTex, USA), E-cadherin (1:1000, 610182, BD), FASN (1:1000, GTX109833, GeneTex), FLNA (1:1000, GTX112939, GeneTex), TALIN-1 (1:500, GTX102215, GeneTex), MRE11 (1:1000, PC388, Calbiochem), DDX3 (1:1000, GTX110614, GeneTex), TCP1 theta (CCT8) (1:1000, GTX105725, GeneTex), CDK2 (1:500, 2546, Cell Signaling), CCT1 (1:250, sc-53454, Santa-cruz Biotechnology), HSP90 (1:1000, sc-13119, Santa-cruz Biotechnology), HSPA1A (1:1000, sc-66048, Santa-cruz Biotechnology), FKBP5 (1:1000, GTX113438, GeneTex), α-tubulin (1:1000, MCA78G, Bio-Rad Laboratories Inc), FLAG (1:2000, F3165, Sigma-Aldrich), c-Myc (1:1000, #11667203001, Roche), CCT2 (1:500, sc-374152, Santa-cruz Biotechnology), CCT3 (1:500, sc-271336, Santa-cruz Biotechnology), HA (1:5000, MMS-101R, Covance), LexA (1:1000, sc-7544, Santa-cruz Biotechnology), and Pgk1 (1:10000, 459250, Invitrogen). Secondary antibodies were employed for Western blotting, including anti-mouse and anti-rabbit peroxidase-conjugated antibodies (Jackson ImmunoResearch Inc.) and an anti-rat IgG (H+L) HRP-conjugated antibody (Bethyl Laboratories Inc.).

### Filter trap assay

The filter trap assay was performed as previously described (64, 65). Cells were harvested and lysed with Filter Trap lysis buffer (1% Triton X-100 in 1x PBS, pH 7.4) containing 1 mM PMSF and Complete EDTA-free Protease Inhibitor Cocktail (Roche, Basel, Switzerland), followed by brief sonication. The protein concentration was determined by the Bio-Rad Protein Assay (Bio-Rad Laboratories). Before filtering, the samples were diluted to a final concentration of 1 μg/ml with filter trap lysis buffer containing 1% sodium dodecyl sulfate. The samples were then filtered through a 0.2 μm nitrocellulose membrane, using a 96-well dot-blot apparatus (Bio-Rad Laboratories). Before filtration, the membranes were immersed in Rinse buffer (1% SDS in 1X PBS, pH 7.4). Filter dots were washed once with 0.05% TBST (150 mM NaCl, 0.05% Tween 20, 20 mM Tris-HCl, pH 7.4). Proteins trapped by the filter were detected by immunoblotting. Chemiluminescence was quantified using the ImageJ software.

### Western blotting

Whole proteins were extracted and resolved by SDS-PAGE and transferred to a polyvinylidene difluoride membrane (Millipore). The membrane was blocked in 3% nonfat milk at room temperature for 1 h, followed by incubation with appropriate primary antibodies at 4 °C overnight. After overnight incubation with the primary antibody, the membrane was washed three times with 0.05% TBST (150 mM NaCl, 0.05% Tween 20, 20 mM Tris-HCl, pH 7.4). Next, the membrane was hybridized with the secondary antibodies for 1 h. Finally, signals were developed using LuminataTM Crescendo Western HRP Substrate (Millipore). The image was quantified by the ImageJ software.

### Co-IP

1 × 10^6^ HEK293T cells were seeded in a 10-cm dish for 24 h, and 4 μg of plasmids were transfected for 48 h. Cells were harvested and lysed in immunoprecipitation (IP) buffer (20 mM Tris-HCl, pH 7.5, 130 mM NaCl, 20 mM Na_2_MoO_4_, 1 mM EDTA, 10% glycerol, 0.5% Triton X-100), supplemented with Complete EDTA-free Protease Inhibitor Cocktail and PhosSTOP Phosphatase Inhibitors (Roche). Cell lysates (900 μg in 450 μl) were incubated for 2 h with anti-c-Myc antibody (1 μg, #11667203001, Roche) or anti-FLAG antibody (2 μg, F3165, Sigma) at 4 °C. Immunocomplexes were then isolated with protein G Mag Sepharose Xtra magnetic beads (Cytiva) for 2 h at 4 °C. After extensive washing with wash buffer (20 mM Tris-HCl, pH 7.5, 150 mM NaCl) three times, the bound proteins were eluted with 40 μl of 2× SDS sample buffer (250 mM Tris, pH 6.8, 10% SDS, 0.25% bromophenol blue, 50% sucrose, 0.5 M 2-mercaptoethanol). Precipitates were then analyzed by Western blotting with appropriate antibodies.

### Cell fractionation analysis

Cell fractionation was conducted as previously described (66). HCT116 cells expressing shFKBP4 were harvested by washing twice with ice-cold PBS. Cells were lysed in 200 μl of Triton lysis buffer (150 mM NaCl, 25 mM Tris-HCl, pH 7.5, 1% Triton X-100, 1 mM EDTA, 20 mM NaF, 1 mM PMSF) supplemented with Complete EDTA-free Protease Inhibitor Cocktail (Roche). A total of 500 μg of cell lysate was centrifuged at 20,000*g* for 30 min at 4 °C, and the supernatant was collected. To ensure complete removal of residual supernatant, pellets were washed with 400 μl of Triton lysis buffer and centrifuged again under the same conditions. The resulting pellets were resuspended in 200 μl of Triton lysis buffer. The Triton-soluble and -insoluble fractions were supplemented with 5× SDS sample buffer and heated at 100 °C for 10 min. The Triton-insoluble fraction was further probe-sonicated and boiled again for 10 min to ensure homogeneity. Both fractions were then subjected to Western blot analysis using the appropriate antibodies.

### BioID assay

The appropriate number of HEK293T cells was seeded into 10 cm culture dishes and transfected separately with pcDNA3.1 MCS-BirA (R118G)-HA, pcDNA3.1 FKBP4-BirA (R118G)-HA, pcDNA3.1 FKBP4 F130Y-BirA (R118G)-HA, pcDNA3.1 MCS-BioID2-HA, pcDNA3.1 FKBP4-BioID2-HA, and pcDNA3.1 FKBP4 F130Y-BioID2-HA constructs. After 48 h of incubation, cells were detached using PBS buffer and lysed with 500 μl of RIPA lysis buffer (50 mM Tris-Cl pH 7.5, 150 mM NaCl, 1 mM EDTA, 1% (v/v) NP-40, 0.1% (w/v) SDS, 0.5% (w/v) sodium deoxycholate, 1 mM DTT, 1:500 protease inhibitor cocktail added just before use). The lysates were sonicated three times for 2 seconds each and then kept on ice for 10 min with intermittent vortexing. Subsequently, the lysates were centrifuged at 13,300 rpm at 4 °C for 4 min. The supernatant was collected and mixed with 798 μl ddH_2_O, 2 μl of protein-dye, and 200 μl of protein sample, while the blank contained 800 μl ddH_2_O and 200 μl of protein dye. Absorbance at 595 nm was measured, and protein concentration was calculated using a standard formula. For the liquid chromatography-tandem mass spectrometry (LC-MS/MS) analysis, 5000 μg of protein was taken; otherwise, 1000 μg was used.

To the remaining lysates, RIPA lysis buffer was added to reach a final volume of 1000 μl, and then 20 μl was taken as the input sample and heated at 100 °C for 5 min after adding 20 μl of 2× sample buffer. Streptavidin beads (100 μl) were added to 1.5 ml Eppendorf tubes along with 750 μl RIPA lysis buffer. After 5 min of incubation at room temperature, the supernatant was removed by low-speed centrifugation, and the remaining protein was added to the streptavidin beads. The tubes were then placed on a rotator and incubated overnight at 4 °C. After overnight incubation, the supernatant was removed, and each tube was washed successively with 1.5 ml of wash buffer 1 (2% (w/v) SDS in ddH_2_O), wash buffer 2 (50 mM HEPES pH 7.5, 500 mM NaCl, 1 mM EDTA, 0.1% (w/v) deoxycholic acid, 1% Triton X-100), and wash buffer 3 (10 mM Tris-Cl pH 7.4, 250 mM LiCl, 1 mM EDTA, 0.1% (w/v) deoxycholic acid, 1% (v/v) NP-40), each for 8 min at room temperature with rotation. After each wash, the supernatant was removed by low-speed centrifugation. Finally, 1.5 ml of 50 mM Tris, pH 7.4, was added to each tube, gently mixed, and centrifuged to remove the supernatant. Biotinylated proteins were eluted with 1X SDS-PAGE sample buffer, incubated at room temperature for 5 min, boiled at 95 °C for 5 min, and then collected.

### LC-MS/MS analysis

MS data were acquired on an Orbitrap Fusion mass spectrometer (Thermo Scientific) equipped with an EASY-nLC 1200 system (Thermo Scientific) and an EASY-Spray HPLC column (75 μm I.D. × 150 mm, 3 μm, 100 Å) and ion source (Thermo Scientific). The chromatographic separation was performed using 0.1% formic acid in water as mobile phase A and 0.1% formic acid in 80% acetonitrile as mobile phase B, operated at a flow rate of 300 nl min-1. The LC gradient was employed from 2% buffer B at 2 min to 40% buffer B at 100 min. Electrospray voltage was maintained at 1.8 kV, and the capillary temperature was set at 275 °C. Full MS survey scans were executed in the mass range of m/z 320 to 1600 (AGC target at 5 × 105) with lock mass, resolution of 120,000 (at m/z 200), and maximum injection time of 50 ms. The MS/MS was run in top speed mode with 3s cycles; while the dynamic exclusion duration was set to 60s with a 10-ppm tolerance around the selected precursor and its isotopes. The precursor ion isolation was performed with mass selecting quadrupole, and the isolation window was set to m/z 2.0. Monoisotopic precursor ion selection was enabled, and 1+ charge state ions were rejected for LC-MS/MS. The LC-MS/MS analyses were carried out with the collision-induced dissociation (CID) mode with a collision energy of 35%. The maximum injection time for spectra acquisition was 150 ms, and the automatic gain control (AGC) target values for MS/MS scans were set at 5 × 10^4^.

### Proteomic data analysis

Acquired MS raw data were converted to mgf format by msConvert (version 3.0.18165, ProteoWizard), then analyzed using Mascot (ver. 2.2, Matrix Science Inc., Boston, USA) for MS/MS ion search. The UniProt human revised protein database (Taxonomy ID: 9606) was used in the Mascot search. For database searches, the precursor mass tolerance was set to 10 ppm, and the fragment ion mass tolerance was set to 0.6 Da. Trypsin was assigned as the enzyme, and 2 missed cleavages were allowed. The carbamidomethylation of cysteine was defined as a fixed modification, and the oxidation of methionine was defined as a variable modification. The interacting proteins were determined by overlapping proteins identified from each sample.

### Yeast two-hybrid assay

The two-hybrid assay was conducted as previously described (67). Briefly, bait plasmids (pEG202 empty vector, pEG202-*CCT8*, pEG202-*MRE11*, and pEG202-*DDX3X*) and the prey plasmids (pJG4-5 empty vector and pJG4-5-*FKBP4*) were transformed into yeast two-hybrid strain YEM1α *(MATa his3-11 trp1-1 LEU2::pLexAop-LEU2 ura3-1:: URA3-pLexAop-Gal1-lacZ),* and Trp^+^ and His^+^ cells were selected. Cells were spotted on SC-Trp-His-Leu galactose plates to inspect the bait-prey reciprocity. For Western blot analysis, yeast strains containing bait and prey plasmids were first grown overnight in synthetic complete (SC)-Trp-His, raffinose medium, and then switched to SC-Trp-His, galactose medium for 3.5 h to induce the *GAL1* promoter of pJG4-5. Cells were collected, and then TCA precipitation was conducted to concentrate the protein for Western blot analysis.

## Data availability

All data generated or analyzed during this study are included in this article.

## Supporting information

This article contains supporting information (67).

## Conflict of interest

The authors declare that they have no conflicts of interest with the contents of this article.
