# Supplementary material for: Hsp90 co-chaperone FKBP4 facilitates CCT8 folding and connects Hsp90 to chaperonin-dependent proteostasis
Source: J Biol Chem. 2025 Nov 5;301(12):110914. doi: 10.1016/j.jbc.2025.110914 (PMC12718141; doi:10.1016/j.jbc.2025.110914)
Supplement: Supporting information [file mmc1.pdf]

Supporting information

Hsp90 co-chaperone FKBP4 facilitates CCT8 folding and connects Hsp90 to chaperonin-dependent proteostasis

Yun-Yu Huang, Ya-Lan Chang, Yun Chen, Wei-Yu Chiang, Ai-Tao Chiang, Pang-Hung Hsu, Shu-Chun Teng

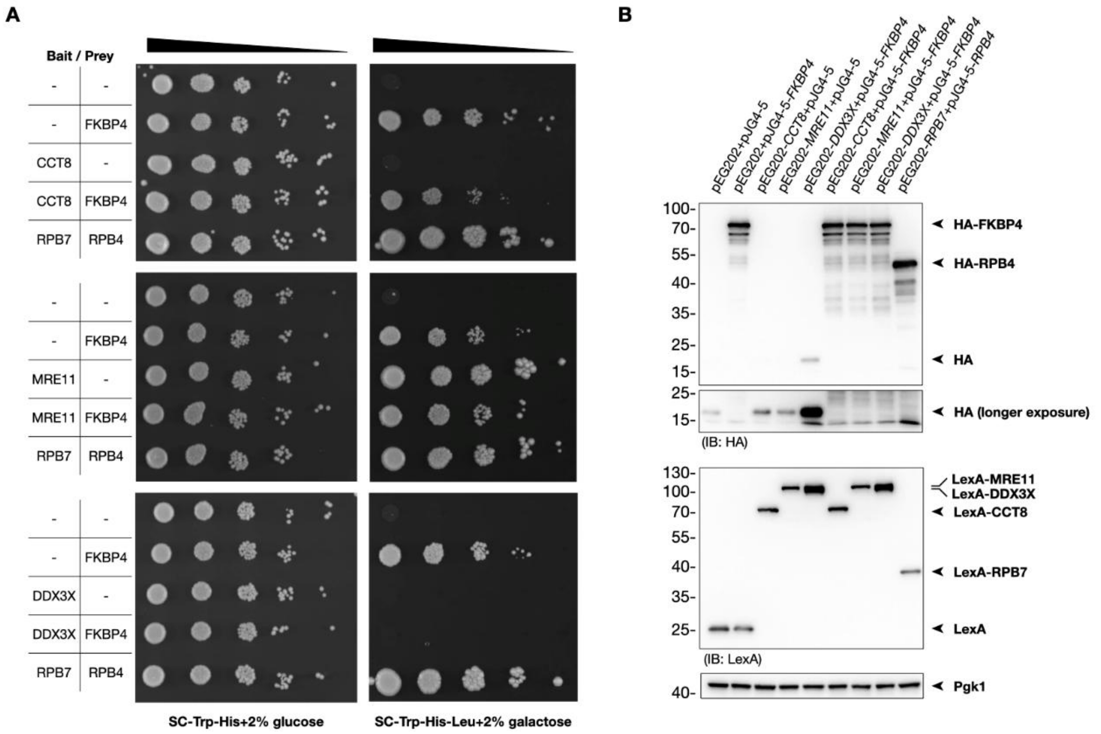

Figure S1. The yeast two-hybrid assay cannot detect the potential interaction between FKBP4 and selected candidate proteins.

(**A**) Cells expressing pEG202 and pJG4-5 empty vector, prey only (FKBP4), bait only (CCT8, MRE11, or DDX3X), and co-expressed bait and prey were spotted in 10-fold dilutions on SC-Trp-His-glucose plates for cell count control and on SC-Trp-His-Leu galactose plates for interaction. Cells expressing Rpb7 and Rpb4, two subunits of RNA polymerase II, were used as a positive control (66). (**B**) Protein lysates were collected after 3.5 hours of galactose induction, and the fusion proteins were examined by Western blot analysis using the indicated antibodies.

```
FKBP4 (H.sapiens)    MTAEEMKATESGAQSAPLPMEGVDISPKQDEGVLKVIKREGTGTEMPMIGDRVFVHYTGW 60
FKBP5 (H.sapiens)    MTTDEGAKNNEESPTATVAEQGEDITSKKDRGVLKIVKRVGNGEETPMIGDKVYVHYKGK 60

FKBP4 (H.sapiens)    LLDGTKFDSSLDRKDKFSFDLGKGEVIKAWDIAIATMKVGEVCHITCKPEYAYGSAGSPP 120
FKBP5 (H.sapiens)    LSNGKKFDSSHDRNEPFVFSLGKGQVIKAWDIGVATMKKGEICHLLCKPEYAYGSAGSLP 120

FKBP4 (H.sapiens)    KIPPNATLVFEVELFEFKGEDLTEEEDGGIIRRIQTRGEGYAKPNEGAIVEVALEGYYKD 180
FKBP5 (H.sapiens)    KIPSNATLFFEIELLDFKGEDLF--EDGGIIRRTKRKGEGYSNPNEGATVEIHLEGRCGG 178

FKBP4 (H.sapiens)    KLFDQRELRFEIGEGENLDLPYGLERAIQRMEKGEHSIVYLKPSYAFGSVGKEKFQIPPN 240
FKBP5 (H.sapiens)    RMFDCRDVAFTVGEGEDHDIPIGIDKALEKMQREEQCILYLGPRYGFGEAGKPKFGIEPN 238

FKBP4 (H.sapiens)    AELKYELHLKSFEKAKESWEMNSEEKLEQSTIVKERGTVYFKEGKYKQALLQYKKIVSWL 300
FKBP5 (H.sapiens)    AELIYEVTLKSFEKAKESWEMDTKEKLEQAAIVKEKGTVYFKGGKYMQAVIQYGKIVSWL 298

FKBP4 (H.sapiens)    EYESSFSNEEAQKAQALRLASHLNLAMCHLKLQAFSAAIESCNKALELDSNNEKGLFRRG 360
FKBP5 (H.sapiens)    EMEYGLSEKESKASESFLLAAFLNLAMCYLKLREYTKAVECCDKALGLDSANEKGLYRRG 358

FKBP4 (H.sapiens)    EAHLAVNDFELARADFQKVLQLYPNNKAAKTQLAVCQQRIRRQLAREKKLYANMFERLAE 420
FKBP5 (H.sapiens)    EAQLLMNEFESAKGDFEKVLEVNPQNKAARLQISMCQKKAKEHNERDRRIYANMFKKFAE 418

FKBP4 (H.sapiens)    EENKAKAEASSGDHPTDTEMKEEQKSNTAGSQSQVETEA------ 459
FKBP5 (H.sapiens)    QDAKEEANKAMGKKTSEGVTN------EKGTDSQAMEEEKPEGHV 457
```

Figure S2. Sequence alignment of FKBP4 and FKBP5.

The amino acid sequences of FKBP4 and FKBP5 were aligned using Uniprot. The conservation of residues is labeled gray.

Fig 1C

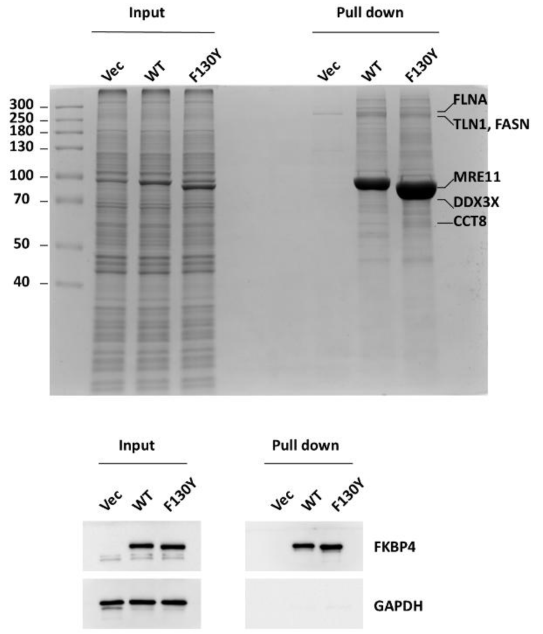

Fig 1D

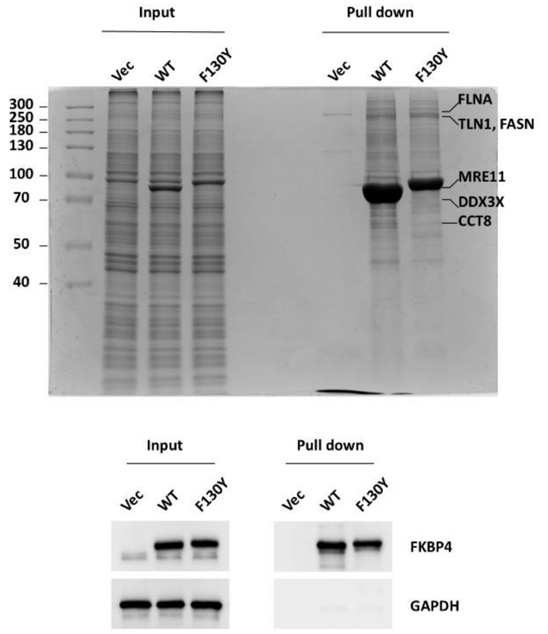

Fig 3A

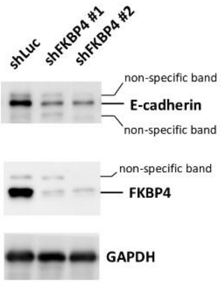

Fig 3B

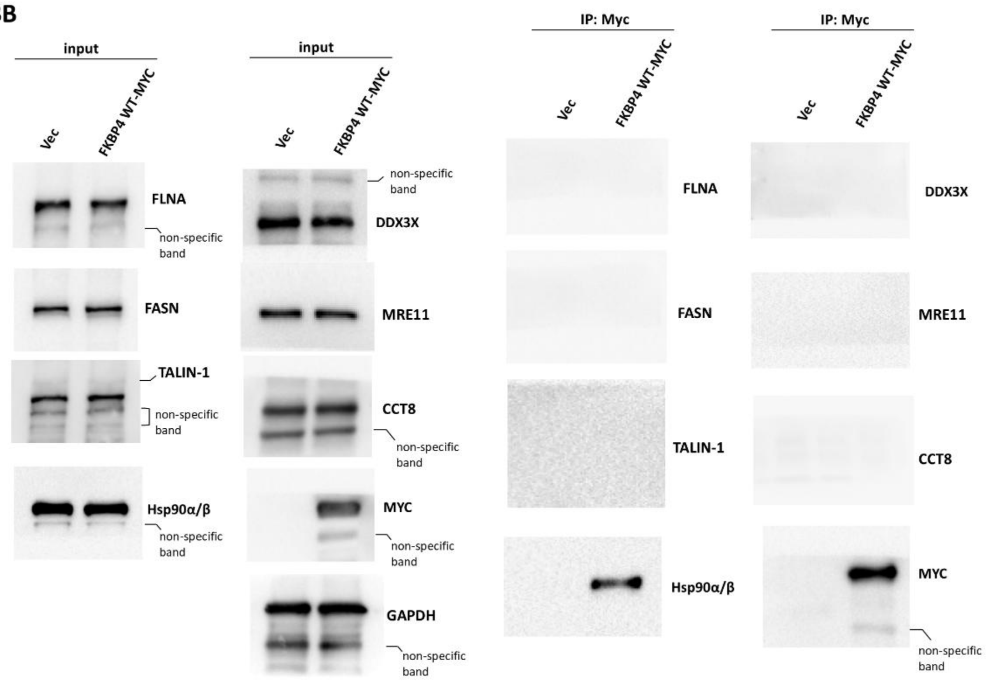

Fig 3C

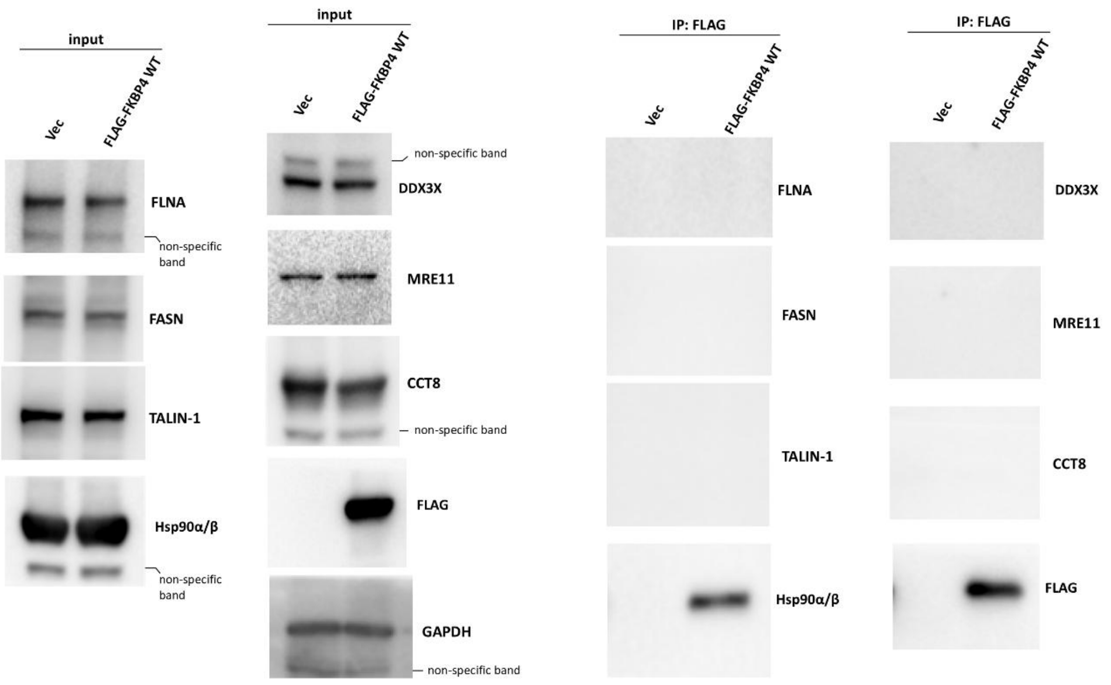

Fig 3D

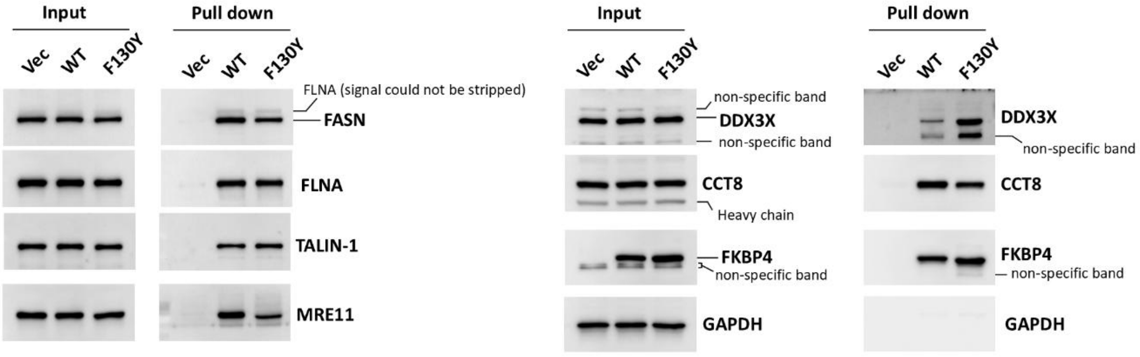

Fig 3E

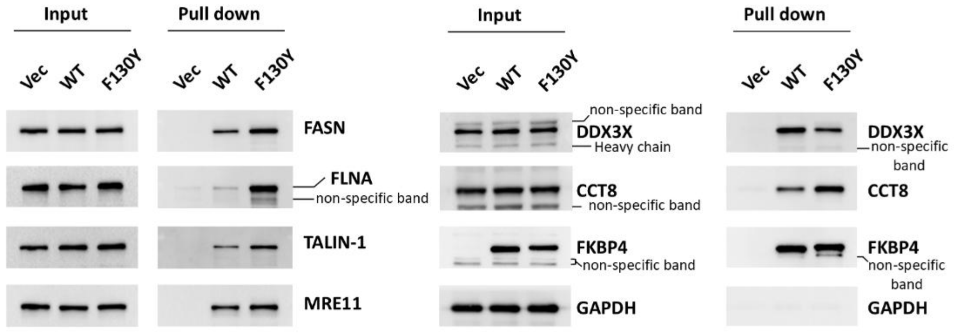

Fig 4A

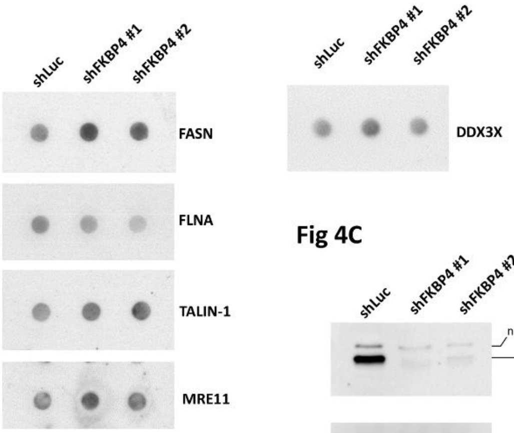

Fig 5A

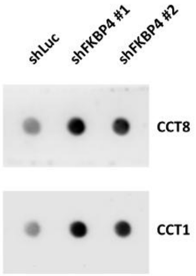

Fig 4C

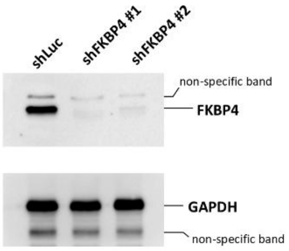

Fig 5C

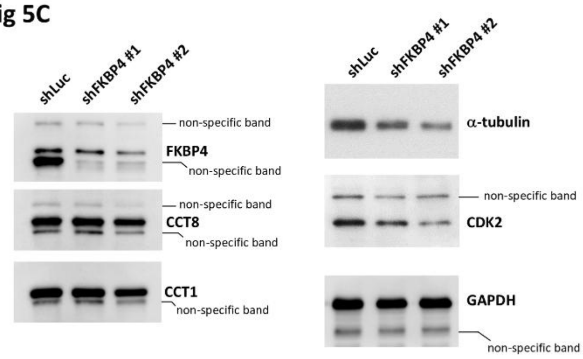

Fig 5E

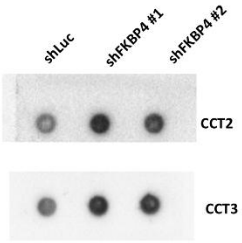

Fig 5G

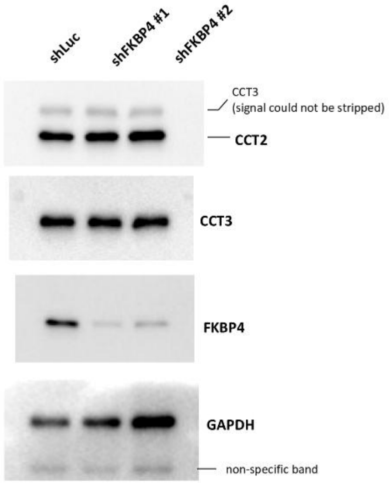

Fig 5I

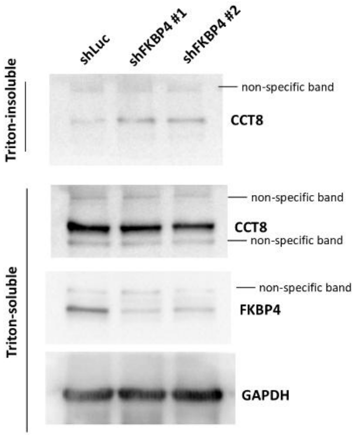

Fig 5L

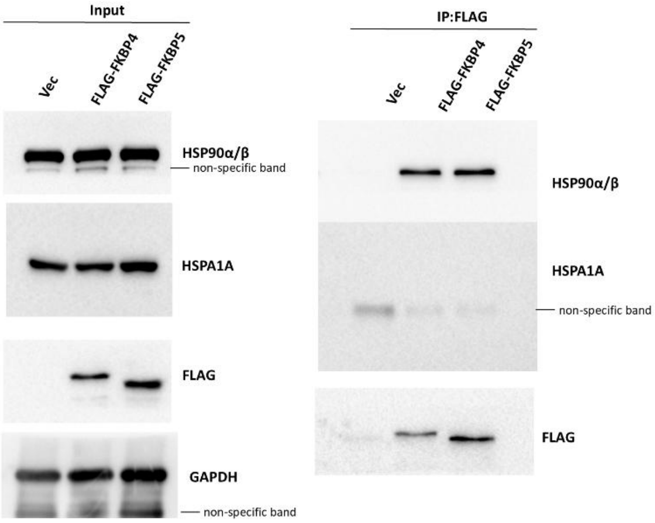

Fig 5N

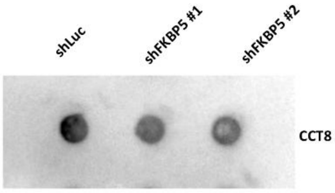

Fig 5P

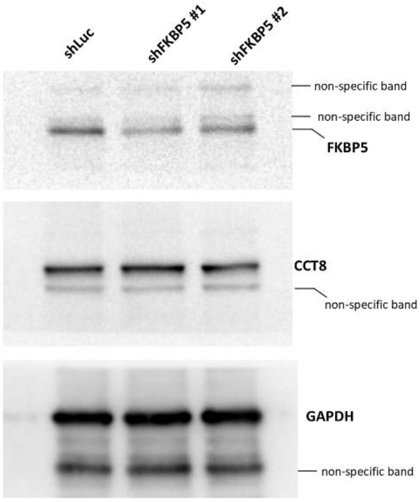

Fig 5R

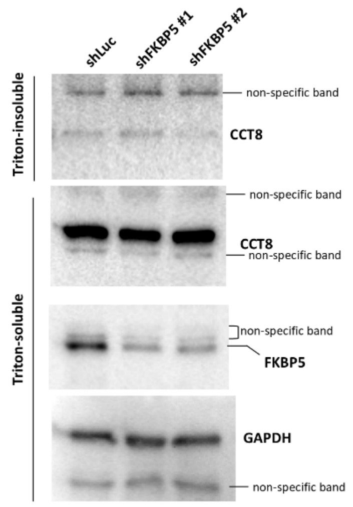

Fig 5T

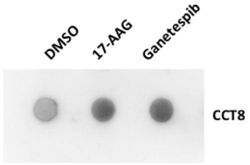

Fig 5V

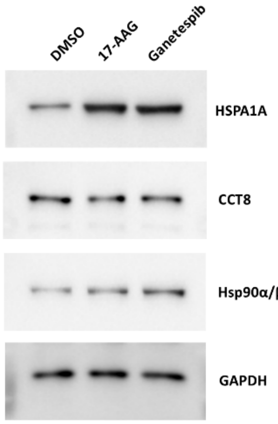

Fig S1 B

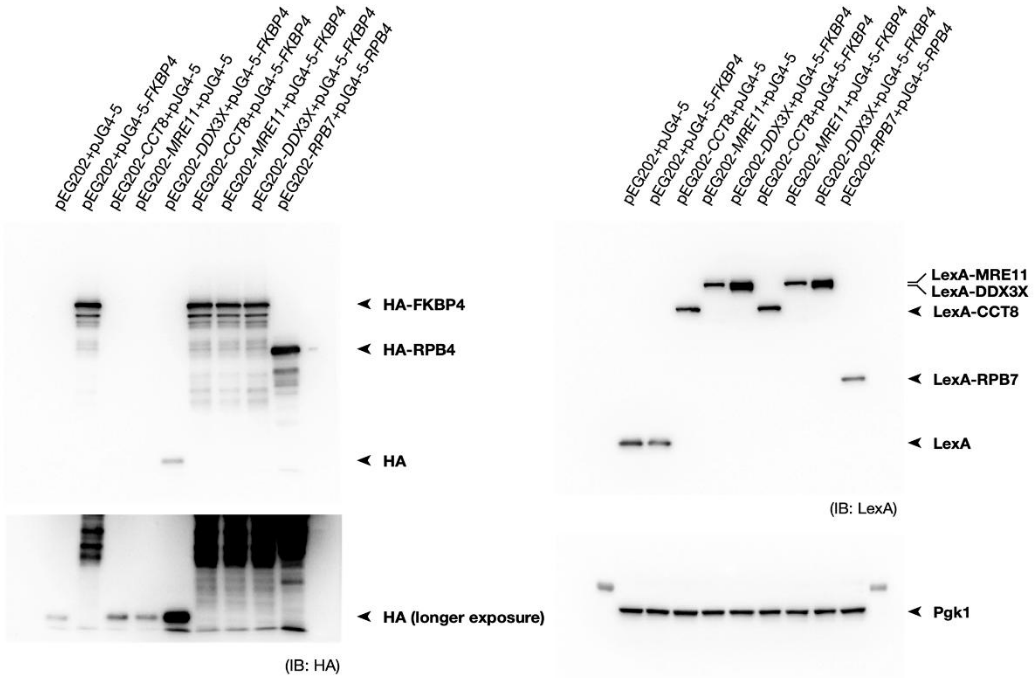

Figure S3. The images of full blots.

The full blot images in this study, including Coomassie blue and Western blot data, are displayed.

Supplementary File Legends

Supplementary File 1. Table listing FKBP4-interacting proteins identified by mass spectrometry in cells overexpressing FKBP4 WT-BirA-HA, compared to those overexpressing the control vector.

Supplementary File 2. Table listing FKBP4-interacting proteins identified by mass spectrometry in cells overexpressing FKBP4 F130Y-BirA-HA, compared to those overexpressing FKBP4 WT-BirA-HA.

Supplementary File 3. Table listing FKBP4-interacting proteins identified by mass spectrometry in cells overexpressing FKBP4 WT-BioID2-HA, compared to those overexpressing the control vector.

Supplementary File 4. Table listing FKBP4-interacting proteins identified by mass spectrometry in cells overexpressing FKBP4 F130Y-BioID2-HA, compared to those overexpressing FKBP4 WT-BioID2-HA.

Supplementary File 5. Gene ontology data of FKBP4-interacting proteins in cells overexpressing FKBP4 WT-BirA-HA, compared to those overexpressing the control vector. Proteins from quantitative mass spectrometry experiments were assessed for enrichment in Gene Ontology categories.

Supplementary File 6. Gene ontology data of FKBP4-interacting proteins in cells overexpressing FKBP4 F130Y-BirA-HA, compared to those overexpressing FKBP4 WT-BirA-HA. Proteins from quantitative mass spectrometry experiments were assessed for enrichment in Gene Ontology categories.

Supplementary File 7. Gene ontology data of FKBP4-interacting proteins in cells overexpressing FKBP4 WT-BioID2-HA, compared to those overexpressing the control vector. Proteins from quantitative mass spectrometry experiments were assessed for enrichment in Gene Ontology categories.

Supplementary File 8. Gene ontology data of FKBP4-interacting proteins in cells overexpressing FKBP4 F130Y-BioID2-HA, compared to those overexpressing FKBP4 WT-BioID2-HA. Proteins from quantitative mass spectrometry experiments were assessed for enrichment in Gene Ontology categories.

Table S1. Knockdown sequence used in this study.

Oligonucleotide sequences of shRNA	Source	Identifier
pLKO.1-shLuc<br>Target seq.:<br>5’GCGGTTGCCAAGAGGTTCCAT3’	Academia Sinica	TRCN0000072249
pLKO.1-shFKBP4 #1<br>Target seq.:<br>5’GAAGAAGCTCTATGCCAATAT3’	Academia Sinica	TRCN0000151216
pLKO.1-shFKBP4 #2<br>Target seq.:<br>5’GCATGGAGAAAGGAGAACATT3’	Academia Sinica	TRCN0000152554
pLKO.1-shFKBP5 #1<br>Target seq.:<br>5’CGAAGGAGCAACAGTAGAAAT3’	Academia Sinica	TRCN0000000235
pLKO.1-shFKBP5 #2<br>Target seq.:<br>5’CCCTCGAATGCAACTCTCTTT3’	Academia Sinica	TRCN0000000237

Table S2. Primer used in this study.

Primers	Source	Sequence
FKBP4-ClaI-F	IDT	5’GCGAATTCATCGATGACAGCCGA
FKBP4-KpnI-R	IDT	5’GTCGACTGGTACCCTATGCTTCTGTCT
FKBP4-NotI-F	IDT	5’AAGCTTGCGGCCGCATGACAGCCGAG
FKBP4-XhoI-R	IDT	5’TCTAGACTCGAGTGCTTCTGTCTCCAC

Table S3. Plasmids used in this study.

Plasmids	Sources	Identifier
pGEM®-T Easy vector	Promega	Cat# A1360
pcDNA3.1 MCS-BirA (R118G)-HA	Addgene	Cat# 36047
pcDNA3.1 FKBP4-BirA (R118G)-HA	In this research	N/A
pcDNA3.1 FKBP4 F130Y-BirA (R118G)-HA	In this research	N/A
pcDNA3.1 MCS-BioID2-HA	Addgene	Cat# 74224
pcDNA3.1 FKBP4-BioID2-HA	In this research	N/A
pcDNA3.1 FKBP4 F130Y-BioID2-HA	In this research	N/A
pcDNA3.1-myc-his vector	Our lab	N/A
pcDNA3.1- FKBP4-myc-his	Our lab	N/A
pFLAG-CMV vector	Our lab	N/A
pFLAG-CMV-FKBP4	Our lab	N/A
pFLAG-CMV-FKBP5	Our lab	N/A
pCMV-Δ8.91	Academia Sinica	N/A
pMD.G	Academia Sinica	N/A
pEG202-*CCT8*	In this research	N/A
pEG202-*MRE11*	In this research	N/A

pEG202-*DDX3X*	In this research	N/A
pJG4-5-*FKBP4*	In this research	N/A

Table S4. Primary antibodies used in this study.

Antibodies	Source	Identifier
FKBP4	abcam	Cat# ab97306<br>RRID: AB_10680055
GAPDH	GeneTex	Cat# GTX100118<br>RRID: AB_1080976
E-cadherin	BD	Cat# 610182<br>RRID: AB_397581
FASN	GeneTex	Cat# GTX109833<br>RRID: AB_1950258
FLNA	GeneTex	Cat# GTX112939<br>RRID: AB_2036932
TALIN-1	GeneTex	Cat# GTX102215<br>RRID: AB_1952268
DDX3	GeneTex	Cat# GTX110614<br>RRID: AB_2036751
MRE11	Millipore	Cat# PC388-100UG<br>RRID: AB_213676
TCP1 theta (CCT8)	GeneTex	Cat# GTX105725<br>RRID: AB_2036474
CCT1	Santa Cruz	Cat# sc-53454<br>RRID: AB_2303272
CDK2	Cell Signaling	Cat# 2546<br>RRID: AB_2276129
HSP90	Santa Cruz	Cat# sc-13119<br>RRID: AB_675659
FKBP5	GeneTex	Cat# GTX113438<br>RRID: AB_2036474
α-tubulin	Bio-Rad	Cat# MCA78G<br>RRID: AB_325005
FLAG	Sigma-Aldrich	Cat# F3165<br>RRID: AB_259529
c-myc	Roche	Cat# 11667203001<br>RRID: AB_390911
CCT2	Santa Cruz	Cat# sc-374152<br>RRID: AB_10917207

CCT3	Santa Cruz	Cat# sc-271336<br>RRID: AB_10610107
HSPA1A	Santa Cruz	Cat# sc-66048<br>RRID: AB_832518
LexA	Santa Cruz	Cat# sc-7544<br>RRID: AB_627883
HA	Covance	Cat# MMS-101R<br>RRID: AB_291262
Pgk1	Invitrogen	Cat# 459250<br>RRID: AB_2532235

Table S5. Chemicals used in this study.

Chemicals and Reagents	Source	Catalog number
DMEM	Cytiva	SH30022.02
Polybrene	Millipore	TR-1003
Puromycin	Sigma-Aldrich	Cat# P8833
LuminataTM Crescendo Western HRP Substrate	Millipore	Cat# WBLUR0500

Table S6. Commercial assay used in this study.

Critical commercial assays		
T-Pro non-liposome transfection reagent II	T-Pro Biotechnology	Cat# JT97-N002M
Bio-Rad Protein Assay	Bio-Rad	Car# 5000006

Table S7. Software used in this study.

**Software**	**Source**	**Identifier**
ImageJ	National Institutes of Health	https://imagej.nih.gov/ij/
BioRender	N/A	https://biorender.com/
msConvert	ProteoWizard	https://proteowizard.sourceforge.io/tools/msconvert.html
Mascot	Matrix Science Inc.	https://www.matrixscience.com/

Table S8. Experimental instruments used in this study.

Other materials and instruments	Source	Identifier
ChemiDoc™ Imaging System	Bio-Rad	Cat# 12003153
96-well dot-blot apparatus	Bio-Rad	Cat# 170-6545
Polyvinylidene difluoride membrane	Millipore	IPVH85R
0.2 μm cellulose acetate membranes	Sterlitech	CA023001
